# Effects of vitamin D on neonatal sepsis: A systematic review and meta‐analysis

**DOI:** 10.1002/fsn3.2003

**Published:** 2020-11-10

**Authors:** Zebenay Workneh Bitew, Teshager Worku, Ayinalem Alemu

**Affiliations:** ^1^ St. Paul’s Hospital Millennium Medical College Addis Ababa Ethiopia; ^2^ School of Nursing and Midwifery College of Health and Medical Sciences Haramaya University Harar Ethiopia; ^3^ Ethipian Public Health Institute Addis Ababa Ethiopia

**Keywords:** 25‐hydroxyvitamin D, neonatal infections, newborn, vitamin D deficiency

## Abstract

Vitamin D deficiency is a major public health concern of pregnant women and neonates worldwide, affecting more than half of neonates. Studies report inconsistent and inconclusive effects of vitamin D treatment on neonatal sepsis. This study aimed to provide conclusive evidence regarding the effect of maternal and cord blood vitamin D levels on neonatal sepsis. Data were retrieved from the electronic database (Web of Science, Scopus, CINAHL [EBSCOhost], ProQuest, EMBASE [Ovid], PubMed, Emcare, MEDLINE [Ovid], and gray literature sources [World cat, Mednar, Google scholar and Google]). Joanna Briggs Institute quality assessment tool was utilized for quality assessment while analysis was performed using Open Meta‐analyst, Comprehensive Meta‐analysis version 3.3.070, and Review Manager version 5.3 software. From the 18 studies included in the study, the overall prevalence of vitamin D deficiency among neonates was 61% (95% CI: 44.3, 77.7); 79.4% (95% CI: 71.6, 87.3) of neonates with sepsis were vitamin D deficient as were 43.7% (23.4, 63.9) of sepsis‐free neonates. Neonates born from mothers with low vitamin D levels were at greater risk of developing neonatal sepsis with a weighed mean difference of −8.57 ng/ml (95% CI: −13.09, −4.05). Similarly, neonates with low cord vitamin D levels were at risk for neonatal sepsis with a mean difference of −8.78 ng/ml (95% CI:‐11.58, −5.99). The incidence of EONS in full‐term newborns was significantly associated with low maternal and cord blood vitamin D levels with weighed mean differences of −11.55ng/ml (95% CI: −17.63, −5.46) & −11.59 ng/ml (95% CI:‐16.65, −6.53), respectively. Low levels of vitamin D both in the cord blood and maternal blood were significantly associated with neonatal sepsis. Hence, vitamin D supplementation for pregnant women and newborns could decrease neonatal sepsis.

## Graphical abstract

This is a systematic review and meta‐analysis about the effect of low level of maternal and cord blood vitamin D levels on neonatal sepsis. It is found that low vitamin D levels are risky for neonatal sepsis.

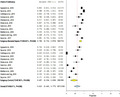

**Key messages**

- Low levels of vitamin D are common in the general neonatal population and are significantly higher in neonates with sepsis.
- Low levels of cord blood and maternal vitamin D are associated with neonatal sepsis.
- Maternal periconceptional and postpartum supplementation of vitamin D could prevent this life‐threatening burden of newborns.

## 1 BACKGROUND

Neonatal sepsis is a systemic infection in the first 28 days of life including bloodstream infections, meningitis, and pneumonia (Moraga & Collaborators, G. C. o. D. 2017). Despite advancements in newborn care, sepsis remains a significant cause of morbidity and mortality globally. The neonatal mortality rate ranges between 11% and 19% of which sepsis contributes to 3 million newborn morbidities (Fleischmann‐Struzek et al., 2018). Globally, sepsis increased neonatal mortality by 1% to 5% and severe sepsis by 9% to 20% (Fleischmann‐Struzek et al., 2018; Hug et al., 2019). The burden is relatively high in Asia and sub‐Saharan Africa (Liu et al., 2015) with the magnitude of neonatal mortality being uneven distributed in sub‐Saharan Africa (29/1000 live births) (You et al., 2015).

Many factors contribute to neonatal sepsis, both maternal and neonatal in origin (Adatara et al., 2019; Oliveira et al., 2016; Hammad & Zainab, 2018; Mugadza et al., 2017; Murthy et al., 2019; Thorne‐Lyman & Fawzi, 2012). An estimated three out of ten neonatal deaths worldwide are thought to be associated with neonatal sepsis secondary to resistant pathogens (Laxminarayan et al., 2016). Annually, around one million newborn deaths are expected to be associated with maternal infections (Black et al., 20166).

Low vitamin D levels in maternal blood during pregnancy and in cord blood are mentioned as risk factors associated with neonatal sepsis. This is explained by vitamin D immune modulation effects which can stimulate the inflammatory mediators and boost the innate immunity (Clancy et al., 2013). It also activates lymphocyte subsets and enhances T‐lymphocyte activation (Youssef et al., 2019). Vitamin D deficiency is common in the general pediatric population and frequently seen in the neonatal period, mainly in the low birthweight and preterm newborns of which neonatal sepsis is more prevalent (McCarthy et al., 2009). Vitamin D deficiency among newborn population ranges from 73% (Khuri‐Bulos et al., 2014) to 94% (Swai et al., 2006), revealing that vitamin D deficiency is a public health problem affecting neonates in several ways. Although low vitamin D levels both in the maternal blood and in the cord blood are associated with newborn infections, there are some studies reporting that this association is insignificant. Thus, these original studies reported inconsistent findings regarding the effects of cord blood and maternal vitamin D levels and the association of neonatal sepsis.

Therefore, the aim of this study was to determine the effects of maternal and newborn cord blood vitamin D levels and the association of neonatal sepsis and to compute the pooled prevalence of cord blood vitamin D deficiency from primary case–control and prospective cohort studies. The pooled estimates were computed using mean and standard deviation of either maternal or cord blood vitamin D levels. The findings of this study will be crucial for policymakers and program planners who are in charge of designing preventive strategies of neonatal sepsis. The findings will have a particular implication for the developing world, where sepsis is the main predictor of newborn deaths (Amare et al., 2019; Cooper, 2014; Rudd et al., 2020).

## 2 METHODS

### 2.1 Searching strategies

The preferred reporting items for systematic review and meta‐analysis (PRISMA) guideline were followed to prepare this systematic review and meta‐analysis (Moher et al., 2009). Searching was conducted by three authors (ZWB, TW, and AA) who were trained in systematic review methods. During the searching process, we consulted a senior librarian working at St. Paul's Hospital Millennium Medical College, Ethiopia. The systematic searches were conducted from electronic and other literature sources in the following databases: Web of Science, Emcare (Ovid), MEDLINE (Ovid), CINAHL (EBSCOhost), EMBASE (Ovid), PubMed, ProQuest and Scopus. Mednar, Google scholar, World cat, and Google were explored for gray literature. In addition, we communicated via personal email exploring unpublished articles as well as information of articles with incomplete reports. The reference lists of included articles were crosschecked for articles which fit the searching criteria and if found treated according to PRISMA guidelines. The searches were conducted using indexed terms, combined with key terms, text words, and search strings taken from the review questions. Advanced search strategies were applied in major databases by three authors (ZWB, TW, and AA) independently. The following were used for searching terms: effect OR impact OR association AND “cord blood” OR “maternal blood” AND vitamin D AND “neonatal sepsis.” The searching terms were checked for being indexed term in each of electronic database before proceeding to the actual search. The Boolean operators such as “AND” or “OR” were used accordingly (See Appendix S1). EndNote X8 reference manager was utilized to manage the searched literature (See Appendix S2).

### 2.2 Inclusion and exclusion criteria

#### 2.2.1 Inclusion criteria

All observational studies (cohort and case–control studies) fulfilling the following criteria were included.

#### 2.2.2 Population

All neonates with neonatal sepsis and their mothers.

#### 2.2.3 Intervention

Vitamin D levels (cord blood and maternal blood vitamin D).

#### 2.2.4 Comparators

Neonates free from neonatal sepsis and their mothers.

#### 2.2.5 Outcome

Neonatal sepsis.

#### 2.2.6 Study designs

Case–control and cohort studies.

#### 2.2.7 Study period

Studies conducted from inception to December 2019 were reviewed and included, accordingly.

### 2.3 Exclusion criteria

The studies fulfilling the following criteria were excluded:

- Articles with irrelevant/incongruent outcomes
- Articles with abstract only and when it was difficult to access the full texts after communicating with the corresponding author(s).
- Articles without mean and standard deviation scores of vitamin D blood levels.

**FIGURE 1:**
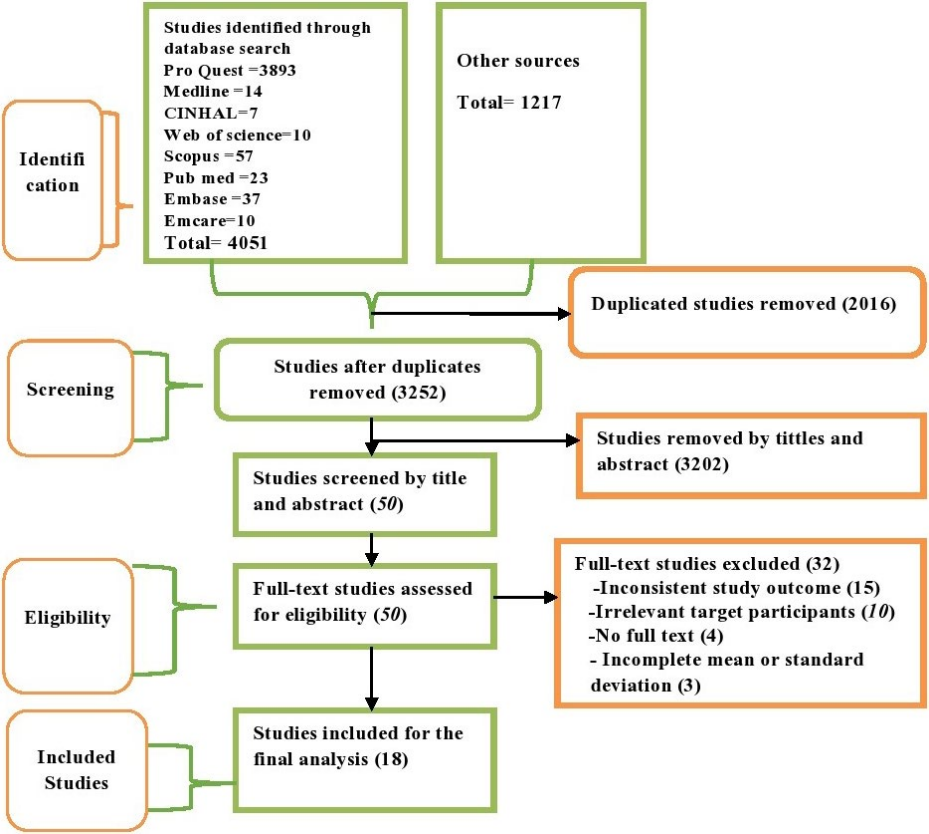
The PRISMA flowchart showing study selection process

Finally, 18 cohort and case–control studies that compared either maternal or cord blood vitamin D levels with neonatal sepsis were included. The details are presented using the PRISMA flow diagram (Figure 1).

### 2.4 Study selection procedure

The EndNote X8 citation manager was used to import studies extracted from several sources, and duplicates were removed. Titles and abstracts were screened by two investigators (ZWB and TW) based on the predefined inclusion criteria. The full texts were collected and evaluated for eligibility for final inclusion considering study subjects, study design, language, methodologic quality, and outcome by two authors (ZWB and AA), independently. Finally, of the eligible articles, the entirety of each article was reviewed by all investigators.

### 2.5 Critical appraisal and data abstraction of the studies

Pretested and structured extraction checklist was used to extract the data, and two investigators (ZWB and TW) performed data extraction. Data (author‐year‐country, study population, gestational age in weeks, study design, sample size, vitamin D deficiency (newborns), neonatal vitamin D level (ng/ml), maternal blood vitamin D levels (ng/ml), and type of neonatal sepsis) were extracted and summarized in Microsoft word 2016 (Table 1). Next, the data were stored in Microsoft excel, 2016 by two authors (ZWB and TW) independently. Finally, the data were cleaned and made ready for the final analysis using the excel spreadsheet. Disagreements between the two authors were resolved by the third author (AA). Joana Briggs Institute checklists of cross‐sectional and cohort studies were used for critical appraisal of the included studies (Munn et al., 2015). Critical appraisal was completed by the two authors (ZWB and AA) independently. The tool has yes, no, unclear, and not applicable options. One was given for yes and zero for all other options. The scores were added up and changed into percentages. In this meta‐analysis, articles with > 50% were included in the final analysis (See Appendix S3). After the critical appraisals were completed, the inter‐rater reliability or inter‐rater agreements were computed prior to the final decision of inclusion of the studies in this systematic review and meta‐analysis. The inter‐rater agreements were computed for both cohort and case–control studies using Cohen's kappa coefficient (κ). The findings revealed that there was substantial agreement (Belur et al., 2018) between the two raters in both case–control (κ = 0.67, *p* ≤ .001) and cohort studies (κ = 0.70, *p* = .024).

**TABLE 1:** Characteristics of studies used to compute the association between low vitamin D levels and neonatal sepsis, 2019

Author(year) country	Study Population		GA(wks)	Study design	Sample	Cord Vit.D deficiency		Neonatal Vitamin D level (ng/ml)		Maternal Vitamin D level		Type of neonatal sepsis	Quality score
	Cases	Controls				Cases (Event/total)	Control (Event/total)	Cases	Controls	Cases	Controls		
Sarwade (2019) Sarwade, 2019, India	30	30	Both	Case–control	60	NA	NA	13.43 ± 3.43	21.53 ± 6.6	NA	NA	EONS	medium
Agrawal et al. (2019) (Agrawal et al., 2019), India	175	50	Term	Case–control	225	151/175	37/50	12.28 ± 6.11	14.88 ± 7.2	NA	NA	LONS	high
Aye et al. (2018) (Aye et al., 2018), Myanmar	40	40	Term	Case–control	80	40/40	26/40	9.82 ± 2.65	18.47 ± 4.37	NA	NA	EONS	medium
Cetinkaya et al. (2015) (Cetinkaya et al., 2015), Turkey	50	50	Term	Cohort	100	42/50	1/50	8.6 ± 3.1	19.0 ± 4.8	22.2 ± 6.8	36.2 ± 1.8	EONS	medium
Dhandai et al. (2018) (Dhandai et al., 2018),India	60	60	Term	Cohort	120	55/60	52/60	15.37 ± 10	21.37 + 9.53	17.87 ± 11.89	23.65 ± l (9.55	LONS	medium
Kumar et al. (2019) (Kumar et al., 2019), India	100	100	Term	Cohort	200	77/100	41/100	NA	NA	NA	NA	EONS	low
Ozdemir and Cag (2019) (Ozdemir & Cag, 2019),Turkey	51	56	Term	Cohort	107	31/51	30/56	11 ± 5.5	13.8 ± 10.6	10.8 ± 5.6	14.9 ± 10	LONS	medium
Gad et al. (2015) (Gad et al., 2015), Egypt	30	30	Term	Case–control	60	15/30	2/30	34.83 ± 18.16	93.20 ± 36.12	NA	NA	EONS	medium
Gamal et al. (2017) (Gamal et al., 2017), Egypt	50	30	Both	Case–control	80	NA	NA	2.56 ± 0.72	9.84 ± 0.88	17 ± 8.28	20.16 ± 8.56	EONS	medium
Tayel et al. (2018) (Tayel et al., 2018), Egypt	40	40	Term	Case–control	80	NA	NA	8.7 ± 0.7	19.1 ± 4.7	21.4 ± 4.8	36.9 ± 4.2	EONS	medium
Cizmeci et al. (2015) (Cizmeci et al., 2015), Turkey	40	43	Term	Case–control	83	28/40	22/43	26.8 ± 18.95	40.5 ± 28.25	NA	NA	EONS	medium
Say et al.(2017) (Say et al., 2017), Turkey	59	41	Preterm	Case–control	100	52/59	7/41	NA	NA	NA	NA	Both	medium
Çekmez et al.(2014) (Çekmez et al., 2014),Turkey	40	20	Term	Case–control	60	NA	NA	49 ± 6.4	35 ± 19	NA	NA	EONS	medium
Dinlen et al.(2016) (Dinlen et al., 2016), Turkey	30	30	Term	Case–control	60	26/30	17/30	NA	NA	NA	NA	LONS	medium
El‐Kader et al.(2018) (El‐Kader et al., 2018), Egypt	25	25	Term	Cohort	50	NA	NA	8.562 ± 2.18	28.55 ± 3.046	22.3 ± 5.047	36.047 ± 1.243	EONS	medium
Mokhtar et al.(2018) (Mokhtar et al., 2018), Egypt	80	80	Term	Case–control	160	52/80	23/80	NA	NA	NA	NA	EONS	medium
Uday et al.(2016) (Uday et al., 2016), India	39	39	Both	Cohort	78	NA	NA	14.69 ± 4.45	26.46 ± 22.01	NA	NA	ENOS	medium
Yang et al.(2016) (Yang et al., 2016), China	78	60	Term	Case–control	138	NA	NA	10 ± 4	14 ± 5	24 + 5	27 + 5	EONS	medium

* Y = yes, *N* = no, U = unclear, NA = not applicable, <60% = low, 60%–80% = medium, >80% = high quality, GA = gestational age, WKS = weeks, Vit.D = vitamin D.

### 2.6 Summary measures

The primary outcome of this systematic review and meta‐analysis was the weighed mean difference of vitamin D levels among neonates with neonatal sepsis and their sepsis‐free neonate counterparts. The pooled prevalence of vitamin D deficiency among neonates with sepsis and control groups was the second outcome. The vitamin D level was measured from both maternal blood and newborn cord blood. The mean vitamin D levels were reported in ng/ml with standard deviations. Then, mean differences were computed from vitamin D levels of both maternal blood and newborn blood. This was used to verify the association between vitamin D levels and neonatal sepsis among neonates. In this study, cases were neonates with sepsis and the counterpart sepsis‐free neonates were considered the control groups. The pooled estimates were presented with 95% CI. The effect sizes were pooled mean differences of vitamin D levels and the prevalence of vitamin D deficiency among neonates with sepsis and the control groups.

### 2.7 Assessment of certainty in the findings and data analysis

The extracted data were exported to OpenMeta‐analyst software to compute the pooled prevalence of cord blood vitamin D deficiency. Review Manager Software was used to analyze the effects of maternal blood and cord blood vitamin D levels on neonatal sepsis. Comprehensive meta‐analysis (CMA) version 3.3.070 software was used to identify publication bias. The pooled estimates were presented using forest plot. The I^2^ test statistic was used to identify heterogeneities between the included studies. The I^2^ values were interpreted as 25% (low), 50% (medium) and 75% (high) heterogeneity. In this study, heterogeneity was declared and justified when *I* ^2^ ≥ 50% with a *p*‐value of ≤0.05. Subgroup and sensitivity analyses were performed to identify the source of heterogeneity and to minimize it. Besides, fixed and random‐effect models were used alternatively in the analyses. Significant heterogeneity was observed between the studies included in computing pooled estimates. Hence, the DerSimonian and Laird random‐effect model (DerSimonian & Laird, 1986) were used to report the pooled estimates. The presence or absence of neonatal sepsis was considered in the subgroup analysis of vitamin D levels in the two groups. Also, an association between maternal and cord blood vitamin D levels with early‐onset neonatal sepsis (EONS) among term neonates was assessed as part of the subgroup analysis. The funnel plot was checked for asymmetry, and Egger's regression test (*p* ≤ .05) (Sterne & Egger, 2001) was used for declaring the presence of publication bias.

## 3 RESULTS

### 3.1 Selection of eligible studies

4,051 articles were retrieved through electronic searching, and 1,217 studies found from gray literature sources. Out of the total articles, 2016 articles were duplicates and removed accordingly. Using titles and abstracts, 3,252 articles were chosen and 3,202 were excluded due to the fact they were not consistent with the study criteria. Next, full texts of the 50 articles were reviewed for eligibility and 32 studies were excluded due to incomplete or inconsistent outcome and study population. Of the excluded studies, 15 (Abbasian et al., 2016; Belderbos et al., 2011; Boskabadi et al., 2019; Camargo et al., 2011; Gharehbaghi et al., 2018; Karras et al., 2016; Liao et al., 2016; Magnus et al., 2013; Mohamed Hegazy et al., 2018; Nageshu et al., 2016; Panda et al., 2019; Puthuraya et al., 2018; Youssef et al., 2019; Zhang et al., 2019; Zheng et al., 2018) were excluded due to irrelevant/incongruent outcomes and ten studies (Ahmed et al., 2015; Binks et al., 2016; Inamo et al., 2011; Khakshour et al., 2015; Łuczyńska et al., 2014; Mansy et al., 2019; McNally et al., 2009; Mohamed & Al‐Shehri, 2013; Roth et al., 2009; Wayse et al., 2004) were excluded due to differences in study participants. The others were excluded because of incompleteness of the mean or standard(Grant, 2010; Prasad et al., 2018; Pulmano, 2018; Tekgunduz et al., 2015) and the absence of full texts(Das et al., 2016; Saboute et al., 2019; Seliem et al., 2016). Finally, a total of 18 articles (Agrawal et al., 2019; Aye et al., 2018; Çekmez et al., 2014; Cetinkaya et al., 2015; Cizmeci et al., 2015; Dhandai et al., 2018; Dinlen et al., 2016; El‐Kader et al., 2018; Gad et al., 2015; Gamal et al., 2017; Kumar et al., 2019; Mokhtar et al., 2018; Ozdemir & Cag, 2019; Sarwade, 2019; Say et al., 2017; Tayel et al., 2018; Uday et al., 2016; Yang et al., 2016) were included in this systematic review and meta‐analysis (Table 1 and Figure 1).

### 3.2 Characteristics of the original studies

In the current study, 12 case–control (Agrawal et al., 2019; Aye et al., 2018; Çekmez et al., 2014; Cizmeci et al., 2015; Dinlen et al., 2016; Gad et al., 2015; Gamal et al., 2017; Mokhtar et al., 2018; Sarwade, 2019; Say et al., 2017; Tayel et al., 2018; Yang et al., 2016) and six cohort (Cetinkaya et al., 2015; Dhandai et al., 2018; El‐Kader et al., 2018; Kumar et al., 2019; Ozdemir & Cag, 2019; Uday et al., 2016) studies were included. Out of the included studies, six were from Turkey (Çekmez et al., 2014; Cetinkaya et al., 2015; Cizmeci et al., 2015; Dinlen et al., 2016; Ozdemir & Cag, 2019; Say et al., 2017), 10 studies were from Egypt (El‐Kader et al., 2018; Gad et al., 2015; Gamal et al., 2017; Mokhtar et al., 2018; Tayel et al., 2018) and India (Agrawal et al., 2019; Dhandai et al., 2018; Kumar et al., 2019; Sarwade, 2019; Uday et al., 2016). The rest were conducted in Myanmar (Aye et al., 2018) and China (Yang et al., 2016). Although articles were searched from inception to 2019, articles published from 2014 to 2019 were eligible for the final analysis of this study. Regarding the quality scores of the studies, most had medium quality (Aye et al., 2018; Çekmez et al., 2014; Cetinkaya et al., 2015; Cizmeci et al., 2015; Dhandai et al., 2018; Dinlen et al., 2016; El‐Kader et al., 2018; Gad et al., 2015; Gamal et al., 2017; Mokhtar et al., 2018; Ozdemir & Cag, 2019; Sarwade, 2019; Say et al., 2017; Tayel et al., 2018; Uday et al., 2016; Yang et al., 2016), whereas one was classified under the low (Kumar et al., 2019) and high (Agrawal et al., 2019) quality category each.

### 3.3 The prevalence of vitamin D deficiency among neonates

**FIGURE 2:**
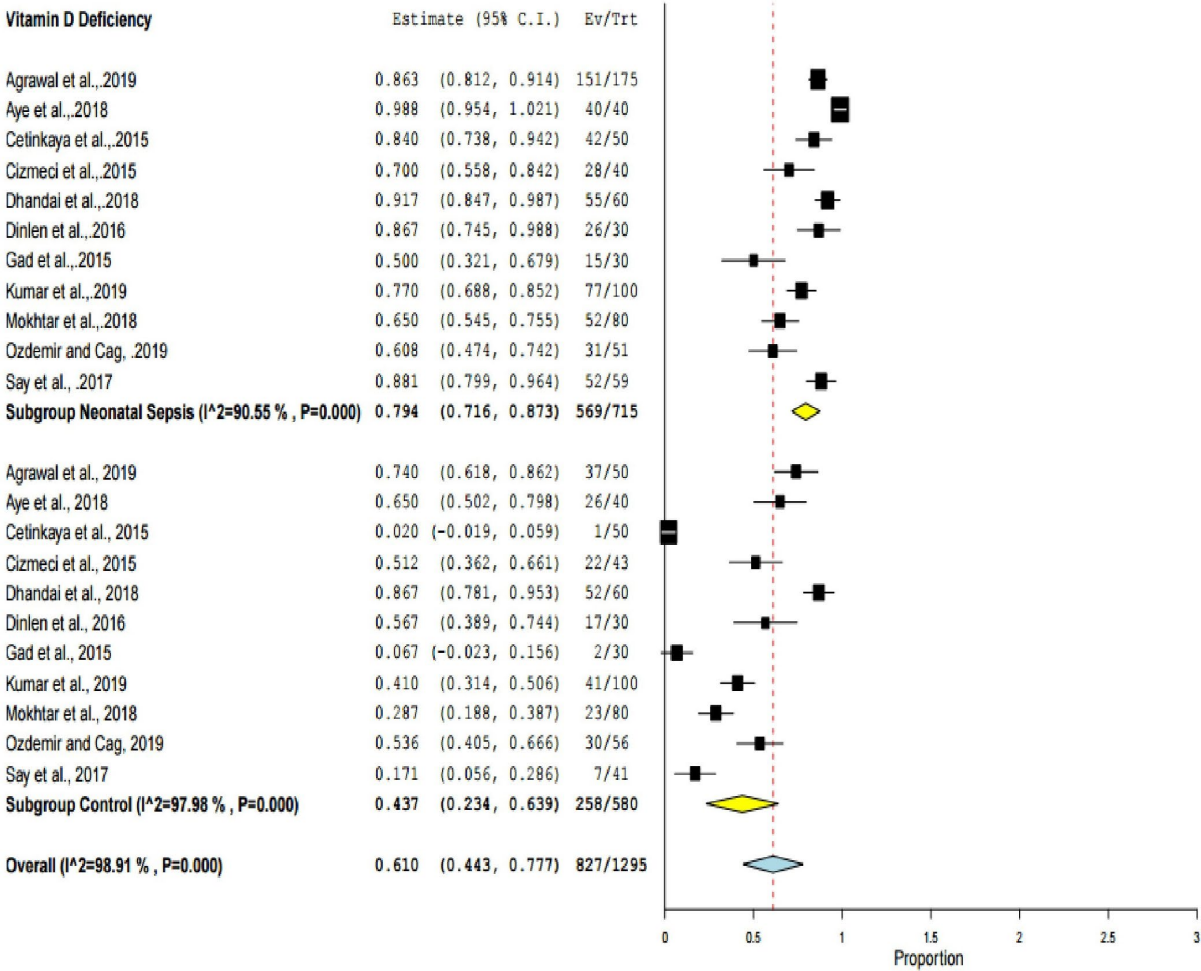
The pooled prevalence of vitamin D deficiency among neonates, 2019

In the present study, vitamin D deficiency was declared when cord blood was less than 20ng/ml (Chang & Lee, 2019). Eleven studies (Agrawal et al., 2019; Aye et al., 2018; Cetinkaya et al., 2015; Cizmeci et al., 2015; Dhandai et al., 2018; Dinlen et al., 2016; Gad et al., 2015; Kumar et al., 2019; Mokhtar et al., 2018; Ozdemir & Cag, 2019; Say et al., 2017) were eligible to compute the pooled vitamin D deficiency among neonates with sepsis and the control groups. From the selected studies, vitamin D deficiency among neonates with neonatal sepsis ranged from 50% (Gad et al., 2015) to 98.8% (Aye et al., 2018), whereas vitamin D deficiency among the controls ranged from 2% (Cetinkaya et al., 2015) to 86.7% (Dhandai et al., 2018). In this meta‐analysis, the pooled estimate of vitamin D deficiency among neonates was 61% (95% CI: 44, 77). In the subgroup analysis, 79.4% (95% CI: 71, 87) of neonates with neonatal sepsis and 43.7% (95% CI: 23.4, 63.9) in the control group had vitamin D deficiency. The heterogeneity test for the pooled estimate was significantly high with I^2^ = 98.9% & *p* ≤ .001. Subgroup analysis and sensitivity analysis were performed to identify the source of heterogeneity. However, all studies were found to have similar contributions for heterogeneity among the included studies. The pooled estimates were also computed for both random and fixed models. However, there was remarkable heterogeneity among the included studies. Hence, DerSimonian and Laird random‐effect model was used in this analysis (Figure 2).

### 3.4 Effect of maternal Vitamin D Level on Neonatal Sepsis

**FIGURE 3:**
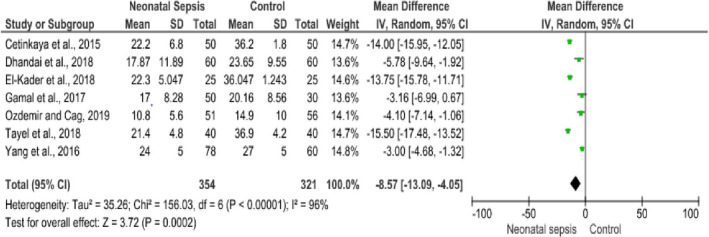
Forest plot showing the association between maternal vitamin D levels with neonatal sepsis, 2019

**FIGURE 4:**
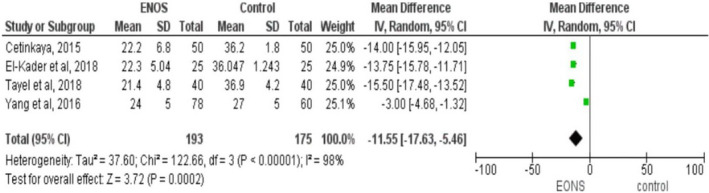
Forest plot showing the association between maternal vitamin D level and early‐onset neonatal sepsis among term neonates, 2019

Seven studies (Cetinkaya et al., 2015; Dhandai et al., 2018; El‐Kader et al., 2018; Gamal et al., 2017; Ozdemir & Cag, 2019; Tayel et al., 2018; Yang et al., 2016) involving 675 participants were included in computing the effect of maternal vitamin D levels on the occurrence of neonatal sepsis. In this study, it was found that there was a significant association between maternal vitamin D levels and neonatal sepsis. Neonates born from mothers having low vitamin D levels were at greater risk of developing neonatal sepsis with a weighed mean difference (MD) of −8.57 ng/ml (95% CI: −13.09, −40.5). The heterogeneity was found to be high (I^2^ = 96%, *p* = .000). Due to this, the DerSimonian and Laird random‐effect model was employed in the final report (Figure 3). In addition, the association of maternal vitamin D levels during pregnancy with the incidence of EONS was checked among term newborns using four eligible studies (Cetinkaya et al., 2015; El‐Kader et al., 2018; Tayel et al., 2018; Yang et al., 2016). It was found that the maternal vitamin D level of newborns with ENOS was significantly lower than the vitamin D levels of control groups (MD=−11.55, 95% CI: −17.63, −5.46 ng/ml; I^2^ = 98% & *p* ≤ .001) (Figure 4).

### 3.5 Effect cord blood vitamin D levels on neonatal sepsis

**FIGURE 5:**
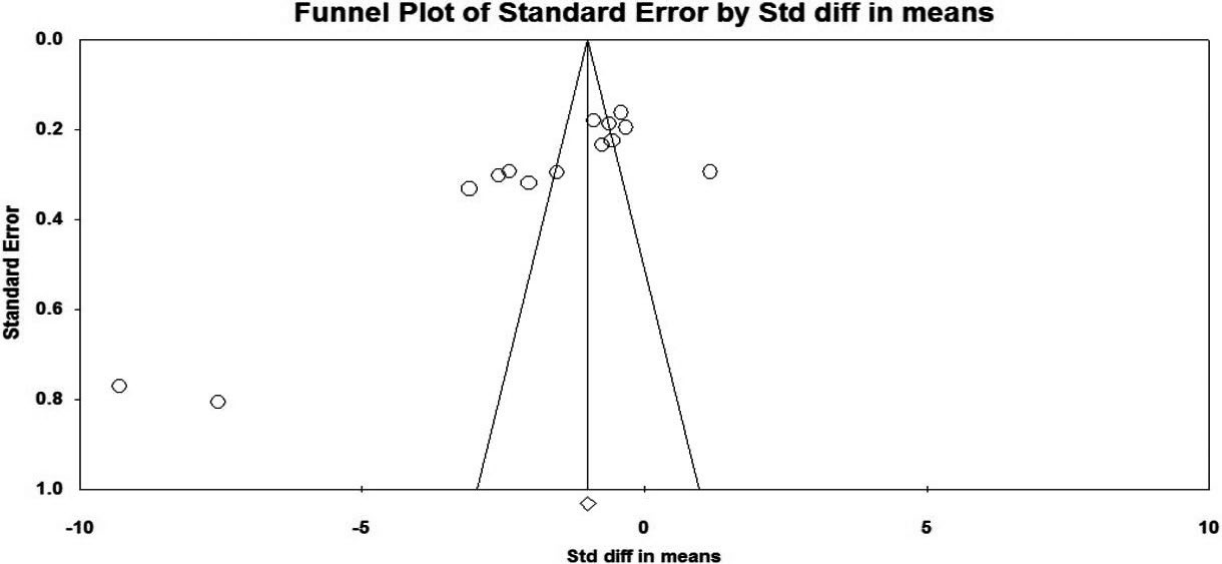
Forest plot showing the association between cord blood Vitamin D levels and neonatal sepsis, 2019

**FIGURE 6:**
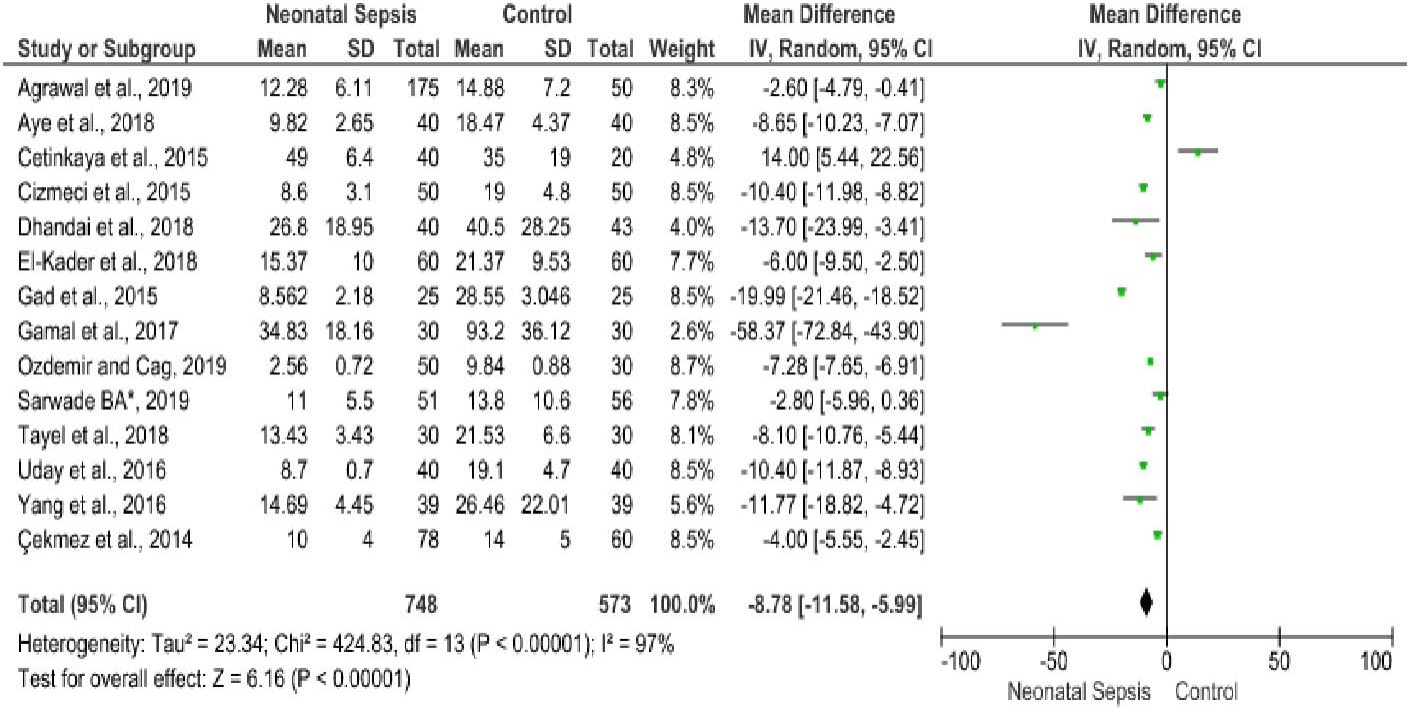
Forest plot showing the association between cord blood vitamin D level and early‐onset neonatal sepsis among term neonates, 2019

**FIGURE 7:**
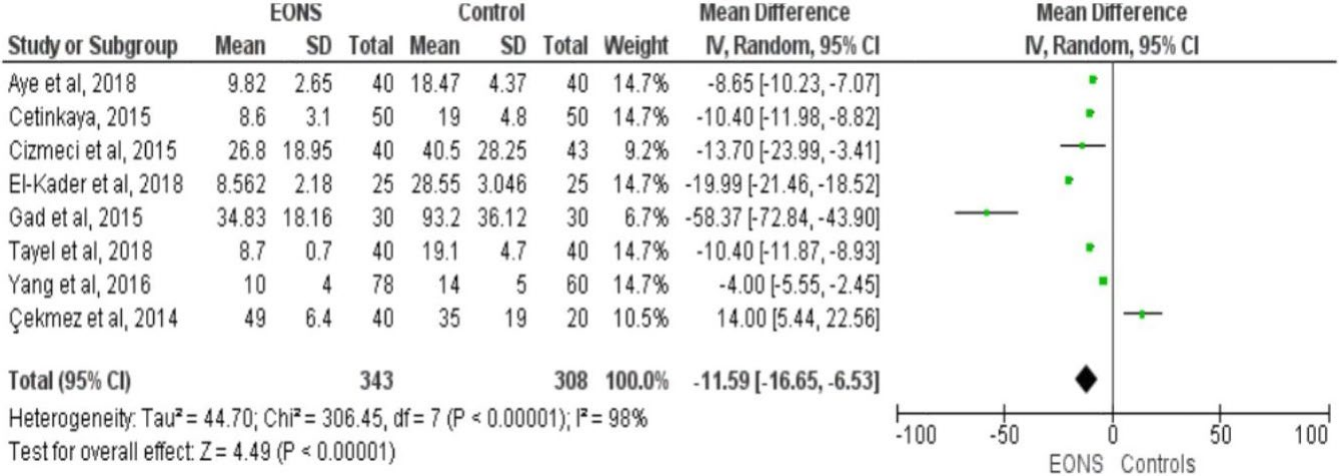
Funnel plot showing publication bias of the included studies for the analysis of the effect of cord Vitamin D level on the neonatal sepsis, 2019

Fourteen studies reported the relationship of cord blood vitamin D level and neonatal sepsis (Agrawal et al., 2019; Aye et al., 2018; Çekmez et al., 2014; Cetinkaya et al., 2015; Cizmeci et al., 2015; Dhandai et al., 2018; El‐Kader et al., 2018; Gad et al., 2015; Gamal et al., 2017; Ozdemir & Cag, 2019; Sarwade, 2019; Tayel et al., 2018; Uday et al., 2016; Yang et al., 2016) involving 748 neonates with sepsis and 573 controls. From these, thirteen studies (Agrawal et al., 2019; Aye et al., 2018; Çekmez et al., 2014; Cizmeci et al., 2015; Dhandai et al., 2018; El‐Kader et al., 2018; Gad et al., 2015; Gamal et al., 2017; Ozdemir & Cag, 2019; Sarwade, 2019; Tayel et al., 2018; Uday et al., 2016; Yang et al., 2016) revealed that low cord blood vitamin D levels were significantly associated with the incidence of neonatal sepsis. In contrast, one study (Cetinkaya et al., 2015) reported the opposite finding with a weighed mean difference of 14 (95% CI: 5.44, 25.5). The overall finding implied that vitamin D levels in neonates’ cord blood with sepsis were lower than the control groups. The pooled MD was −8.78 ng/ml (95% CI: −11.58, −5.99) with remarkable heterogeneity among the included studies (I^2^ = 97%, *p* ≤ .001). Sensitivity and subgroup analyses were conducted to identify the possible sources of heterogeneity among the included studies. Nonetheless, no significant difference was observed in the pooled estimates. Finally, publication bias was checked using a funnel plot (Figure 5). On the inspection of the funnel plot, publication bias was suspected. To confirm the suspicion, Egger's regression test was performed and the result showed publication bias (the intercept (B0) is −11.733, (95% CI: −17.388, −6.078), with t = 4.52, *df* = 12.000. The p‐value (1‐tailed) was 0.00035, and the p‐value (2‐tailed) was 0.0007). Hence, DerSimonian and Laird random‐effect model was used in computing the final MD (Figure 6). Specifically, the association between cord blood vitamin D levels with EONS in term neonates was assessed using eight studies fulfilling the inclusion criteria (Aye et al., 2018; Çekmez et al., 2014; Cetinkaya et al., 2015; Cizmeci et al., 2015; El‐Kader et al., 2018; Gad et al., 2015; Tayel et al., 2018; Yang et al., 2016). Vitamin D levels in cord blood of term neonates with EONS were significantly lower as compared to neonates free from EONS (MD=−11.59, 95%CI: −16.65, −6.53 ng/ml; I^2^ = 98% & *p* ≤ .001) (Figure 7).

## 4 DISCUSSION

The current systemic review and meta‐analysis presented the pooled prevalence of cord blood vitamin D deficiency and the effect of low maternal and cord vitamin D levels on the occurrence of neonatal sepsis. To generate conclusive evidence in this area, prospective cohort and case–control studies were included. A total of 18 studies have been included to compute the respective pooled estimates. The pooled estimates were presented in the random‐effect model due to the presence of considerable heterogeneity among the included studies. The presence of high heterogeneity could be attributed to differences in the study settings, sample size differences, and discrepancies during the measurement of vitamin D levels by the original studies.

Vitamin D levels during pregnancy and the perinatal period are a crucial factor which determines health outcomes of neonates. According to this study, vitamin D deficiency continues to remains high among neonates without much attention to tackle the problem. The pooled prevalence of vitamin D deficiency is high among neonates and significantly higher among neonates with neonatal sepsis. On the other side, vitamin D deficiency among neonates without neonatal sepsis is confirmed to be low. In the present study, the pooled prevalence of vitamin D deficiency among all neonates was 61%. The prevalence was significantly higher (79.4%) among neonates with sepsis as compared to neonates without sepsis (43.7%). This finding was relatively lower than a study conducted in India, where the prevalence of vitamin D deficiency among neonates with sepsis was 91.7% and 86.7% in the control group (sepsis‐free neonates) (Dhandai et al., 2018). However, these findings are higher than a study conducted in Egypt, where 50% and 6.7% of cases and controls were vitamin D deficient, respectively (Gad et al., 2015). The possible elucidation for the discrepancies could be associated with sample size differences, geospatial variations, and differences in the study setting of the original studies.

In this study, vitamin D levels in the maternal blood and cord blood were significantly associated with neonatal sepsis incidence. So that it is possible to infer that neonates born from mothers having a low vitamin D level are at greater risk of developing neonatal sepsis. Similarly, low levels of vitamin D in newborns’ cord blood were found to have a significant association with neonatal sepsis. Despite this, vitamin D is not part of the standard care of pregnant women in many countries. This is supported by a systematic review and meta‐analysis of four studies on the association between vitamin D levels and neonatal early‐onset sepsis (Lee et al., 2018), which revealed that neonatal sepsis was significantly associated with low vitamin D levels in the maternal blood and cord blood. According to this study, the weighted mean difference in vitamin D levels in neonates with early‐onset sepsis and controls was −7.27 ng/ml (95% CI:‐7.62, 6.92). The weighted mean difference in maternal vitamin D levels of neonates with neonatal sepsis was −7.24 ng/ml (95% CI: −8.45, −6.03). Similarly, another systemic review also supports our finding that vitamin D supplementation decreases respiratory tract infections (OR = 0.582, 95% CI: 0.417,0.812) (Charan et al., 2012). Besides, our finding is supported by a recent meta‐analysis finding which showed a significant association between vitamin D deficiency/lower 25(OH)D levels and sepsis in neonates and children (Xiao et al., 2020). A cohort study conducted in India was also in line with these present findings (Behera et al., 2020). Thus, supplementation of vitamin D during pregnancy can increase the mother and the offspring's vitamin D levels. This could boost the immune system of neonates and can decrease neonatal mortality that might be associated with neonatal sepsis (Bi et al., 2018; Karras et al., 2016).

In this study, the relationship between cord blood and maternal vitamin D levels with EONS among term newborns was also analyzed. The likelihood of EONS in term newborns was found to be significantly associated with low levels of both maternal and cord blood vitamin D. The weighed mean difference between maternal vitamin D levels of term newborns with EONS and controls is −11.55 ng/ml. Likewise, the weighed mean difference between cord blood vitamin D levels in term newborns with EONS and controls is −11.59 ng/ml. The possible elucidation is that vitamin D could prevent bacterial infections due to its’ immunomodulation effects which decrease chemokine production, T‐cell activation, and inhibit dendritic cell activation (Karras et al., 2016). Therefore, we recommend periconceptional supplementation of vitamin D especially in African countries where the magnitude of sepsis in neonates is significantly higher (Belachew & Tewabe, 2020; Seale et al., 2013). The studies included in this meta‐analysis are limited to some countries, and thus, additional primary studies could unfold the strong association of vitamin D deficiency with neonatal sepsis and related neonatal disorders.

### 4.1 Strengths and Limitations

The strengths of this study were multiple databases were explored, and all possible analyses were done to compute the pooled estimates. To our knowledge, this study is the first of its type in estimating the pooled vitamin D levels in both maternal blood and cord blood by using a relatively large number of studies globally. Thus, these findings will pave the wave to conduct controlled clinical trials in the future. However, this study has limitations such as differences in the units of measurements, incompleteness of the findings, and in some studies, the vitamin D levels were reported with different measures of central tendency. Hence, some studies were excluded due to those discrepancies and this could affect the pooled estimates of this systematic review and meta‐analysis.

## 5 CONCLUSION

Vitamin D has multiple effects on neonatal health including the prevention of neonatal sepsis. Low levels of vitamin D both in pregnant mothers and in the cord blood of newborns were significantly associated with neonatal sepsis. Specifically, early‐onset neonatal sepsis in term newborns was also associated with low levels of both maternal and cord blood vitamin D. Therefore, it is recommended to supplement vitamin D during pregnancy to decrease sepsis in neonates. To clearly identify the timing, the dosage and mode of vitamin D supplementation, comprehensive randomized controlled trial studies are recommended.

## CONFLICT OF INTEREST

The authors declare that they have no competing interests.

## ETHICS APPROVAL

Not applicable in this study.

## Supporting information

Appendix S1

Appendix S2

Appendix S3

## Data Availability

All important data are included with in the manuscript, and others can be obtained upon request of the corresponding author.
