# Supplementary figures and images for: Effects of vitamin D on neonatal sepsis: A systematic review and meta‐analysis
Source: Food Sci Nutr. 2020 Nov 10;9(1):375–88. doi: 10.1002/fsn3.2003 (PMC7802542; doi:10.1002/fsn3.2003)

Search String

Medline


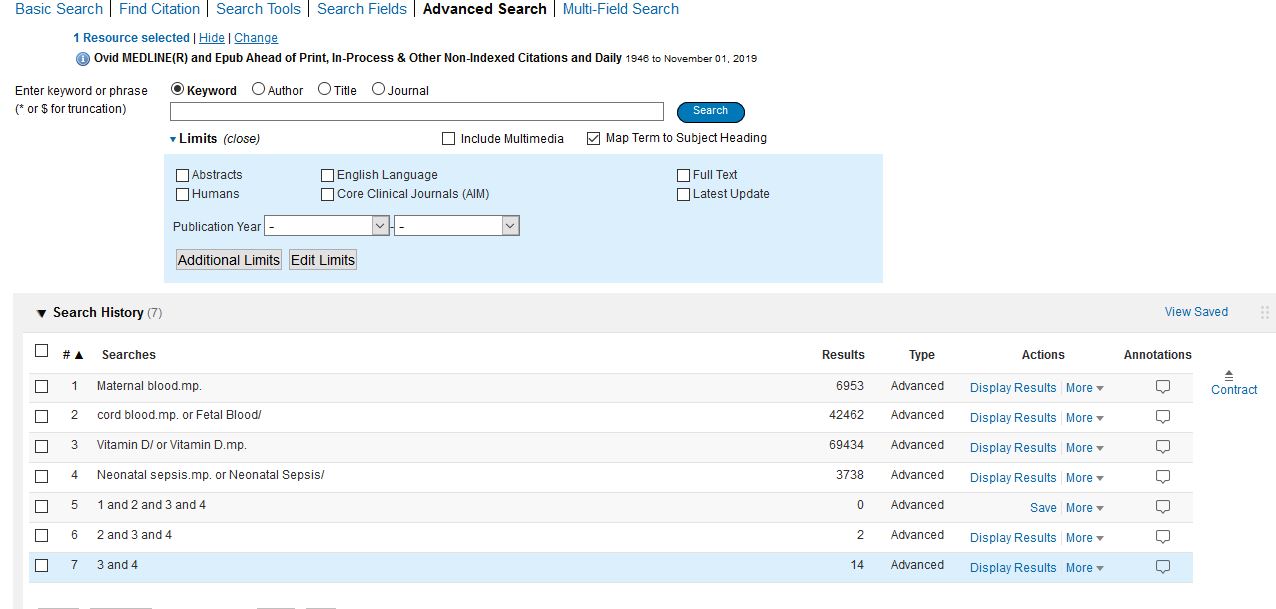


Pubmed


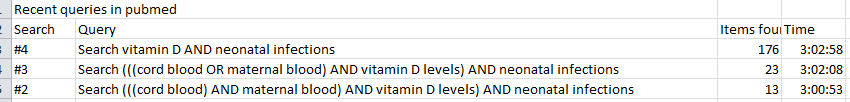


Scopus


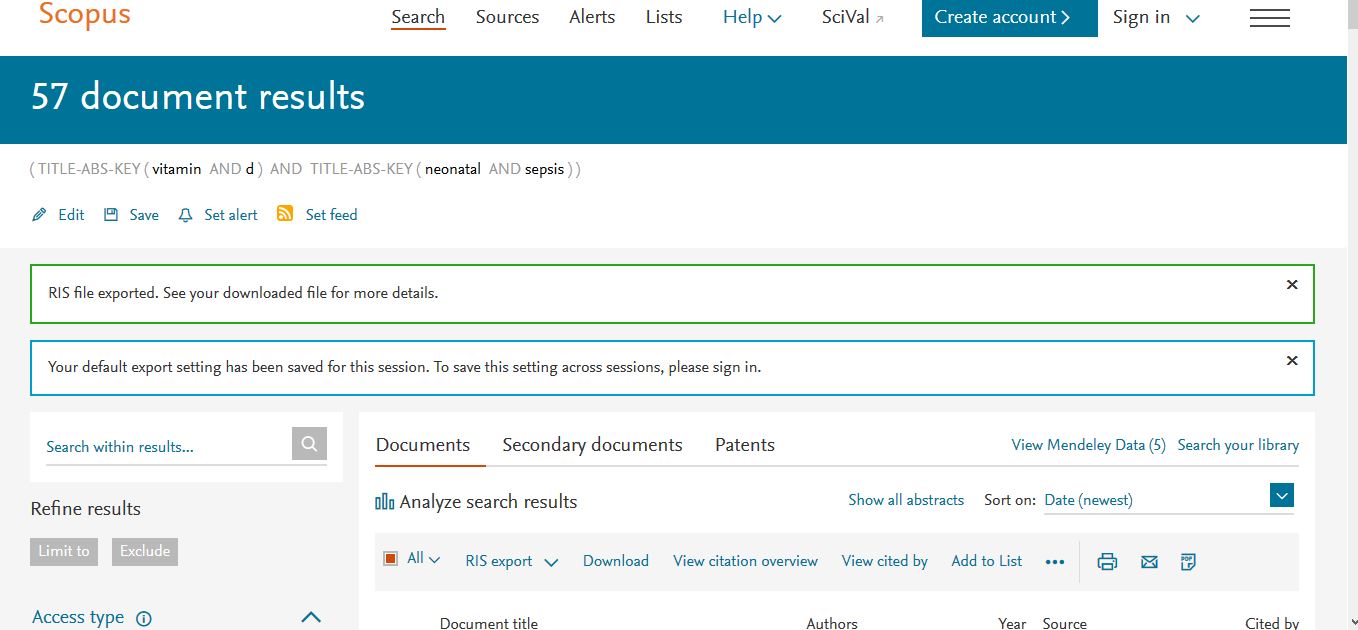


Web of science


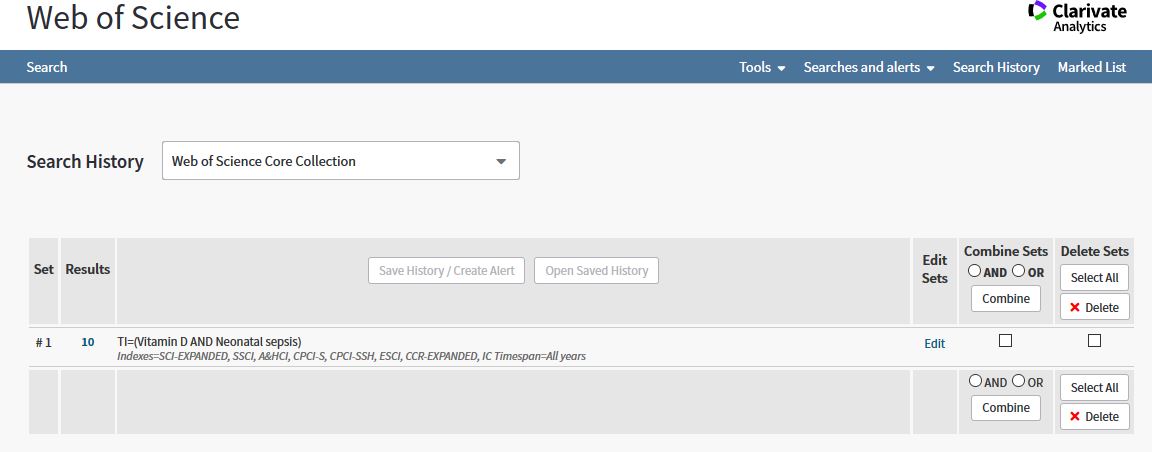


ProQuest


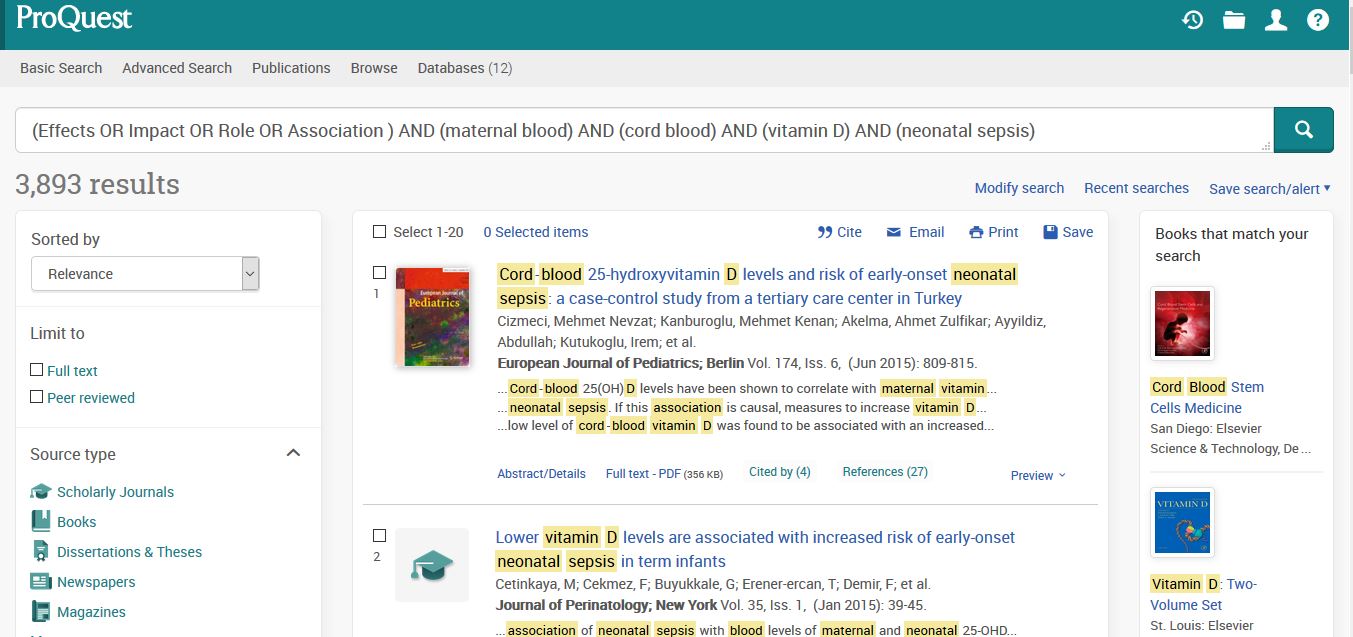

Supplement: Supplementary file 1 — Appendix S1 [file FSN3-9-375-s001.docx]
