# Supplementary material for: Effects of vitamin D on neonatal sepsis: A systematic review and meta‐analysis
Source: Food Sci Nutr. 2020 Nov 10;9(1):375–88. doi: 10.1002/fsn3.2003 (PMC7802542; doi:10.1002/fsn3.2003)
Supplement: Supplementary file 3 — Appendix S3 [file FSN3-9-375-s003.docx]

| Author, year | Q1 | | | | Q2 | | | | Q3 | | | | Q4 | | | | Q5 | | | | Q6 | | | | Q7 | | | | Q8 | | | | Q9 | | | | Q10 | | | | Overall quality result |
| --- | --- | --- | --- | --- | --- | --- | --- | --- | --- | --- | --- | --- | --- | --- | --- | --- | --- | --- | --- | --- | --- | --- | --- | --- | --- | --- | --- | --- | --- | --- | --- | --- | --- | --- | --- | --- | --- | --- | --- | --- | --- |
|  | Y | N | U | NA | Y | N | U | NA | Y | N | U | NA | Y | N | U | NA | Y | N | U | NA | Y | N | U | NA | Y | N | U | NA | Y | N | U | NA | Y | N | U | NA | Y | N | U | NA |  |
| Agrawal et al., 2019 | √ |  |  |  |  | √ |  |  | √ |  |  |  | √ |  |  |  | √ |  |  |  | √ |  |  |  | √ |  |  |  | √ |  |  |  | √ |  |  |  | √ |  |  |  | 9/10 (90%) |
| Aye et al., 2018 | √ |  |  |  | √ |  |  |  | √ |  |  |  | √ |  |  |  |  | √ |  |  |  | V |  |  |  | √ |  |  | √ |  |  |  | √ |  |  |  |  | √ |  |  | 6/10 (70%) |
| Çekmez et al., 2014 | √ |  |  |  | √ |  |  |  | √ |  |  |  |  |  | √ |  | √ |  |  |  |  |  | √ |  | √ |  |  |  | √ |  |  |  | √ |  |  |  |  | √ |  |  | 6/10(60%) |
| Cizmeci et al., 2015 | √ |  |  |  | √ |  |  |  | √ |  |  |  | √ |  |  |  | √ |  |  |  |  |  | √ |  |  | √ |  |  | √ |  |  |  | √ |  |  |  |  | √ |  |  | 7/10 (70%) |
| Dinlen et al., 2016 | √ |  |  |  | √ |  |  |  | √ |  |  |  | √ |  |  |  | √ |  |  |  | √ |  |  |  | √ |  |  |  | √ |  |  |  | √ |  |  |  | √ |  |  |  | 8/10(80%) |
| Gad et al., 2015 | √ |  |  |  | √ |  |  |  | √ |  |  |  | √ |  |  |  | √ |  |  |  |  |  | √ |  |  | √ |  |  | √ |  |  |  | √ |  |  |  |  | √ |  |  | 7 (70%) |
| Gamal et al., 2017 | √ |  |  |  |  | √ |  |  | √ |  |  |  | √ |  |  |  | √ |  |  |  | √ |  |  |  | √ |  |  |  | √ |  |  |  |  | √ |  |  | √ |  |  |  | 8/10(80%) |
| Mokhtar et al., 2018 | √ |  |  |  | √ |  |  |  |  | √ |  |  | √ |  |  |  | √ |  |  |  | √ |  |  |  |  |  | √ |  |  | √ |  |  | √ |  |  |  | √ |  |  |  | 8/10(80%) |
| Sarwade BA*, 2019 | √ |  |  |  | √ |  |  |  |  | √ |  |  | √ |  |  | √ |  |  |  |  |  | √ |  |  |  | √ |  |  | √ |  |  |  | √ |  |  |  |  | √ |  |  | 6/10(60%) |
| Say et al., 2017 | √ |  |  |  |  |  | √ |  |  |  | √ |  | √ |  |  |  | √ |  |  |  |  |  | √ |  |  |  | √ |  | √ |  |  |  | √ |  |  |  | √ |  |  |  | 6/10 (60%) |
| Tayel et al., 2018 | √ |  |  |  | √ |  |  |  | √ |  |  | √ |  |  |  |  | √ |  |  |  |  | √ |  |  |  |  | √ |  | √ |  |  |  | √ |  |  |  | √ |  |  |  | 8/10(80%) |
| Yang et al., 2016 | V |  |  |  | √ |  |  |  | √ |  |  |  | √ |  |  | √ |  |  |  |  |  |  | √ |  |  |  | √ |  | √ |  |  |  |  |  | √ |  | √ |  |  |  | 7/10 (70%) |

**The critical appraisal for Case Control studies**

****Y=yes, N=no, U=unclear, NA=not applicable, <60%=low,60-80%=medium, >80%=high quality***

**The critical appraisal for cohort studies**

| Author, year | Q1 | | | | | Q2 | | | | Q3 | | | | Q4 | | | | Q5 | | | | Q6 | | | | Q7 | | | | Q8 | | | | Q9 | | | | Q10 | | | | Q11 | | | | Overall quality Result |
| --- | --- | --- | --- | --- | --- | --- | --- | --- | --- | --- | --- | --- | --- | --- | --- | --- | --- | --- | --- | --- | --- | --- | --- | --- | --- | --- | --- | --- | --- | --- | --- | --- | --- | --- | --- | --- | --- | --- | --- | --- | --- | --- | --- | --- | --- | --- |
|  | Y | | N | U | NA | Y | N | U | NA | Y | N | U | NA | Y | N | U | NA | Y | N | U | NA | Y | N | U | NA | Y | N | U | NA | Y | N | U | NA | Y | N | U | NA | Y | N | U | NA | Y | N | U | NA |  |
| Cetinkaya et al., 2015 | | √ |  |  |  | √ |  |  |  |  |  | √ |  | √ |  |  |  |  |  | √ |  | √ |  |  |  | √ |  |  |  |  |  | √ |  | √ |  |  |  |  | √ |  |  | √ |  |  |  | 7/11 (63.64%) |
| Dhandai et al., 2018 | | √ |  |  |  | √ |  |  |  |  |  | √ |  | √ |  |  |  |  |  | √ |  | √ |  |  |  | √ |  |  |  |  |  | √ |  | √ |  |  |  | √ |  |  |  | √ |  |  |  | 8/11 (72.73%) |
| El-Kader et al., 2018 | | √ |  |  |  | √ |  |  |  | √ |  |  |  |  | √ |  |  | √ |  |  |  | √ |  |  |  | √ |  |  |  | √ |  |  |  |  | √ |  |  |  | √ |  |  | √ |  |  |  | 8/11(72.73%) |
| Ozdemir and Cag, 2019 | | √ |  |  |  | √ |  |  |  | √ |  |  |  |  | √ |  |  |  | √ |  |  | √ |  |  |  | √ |  |  |  | √ |  |  |  |  |  | √ |  |  |  | √ |  | √ |  |  |  | 7/11 (63.64%) |
| Uday et al., 2016 | | √ |  |  |  | √ |  |  |  | √ |  |  |  |  | √ |  |  |  | √ |  |  | √ |  |  |  | √ |  |  |  | √ |  |  |  |  | √ |  |  |  | √ |  |  | √ |  |  |  | 7/11 (63.64%) |
| Kumar et al., 2019 | | √ |  |  |  | √ |  |  |  | √ |  |  |  | √ |  |  |  |  | √ |  |  | √ |  |  |  | √ |  |  |  | √ |  |  |  |  | √ |  |  |  | √ |  |  |  |  | √ |  | 7/11(63.64%) |

****Y=yes, N=no, U=unclear, NA=not applicable, <60%=low, 60-80%=medium, >80%=high quality***
